# The Many Faces of Interleukin-6: The Role of IL-6 in Inflammation, Vasculopathy, and Fibrosis in Systemic Sclerosis

**DOI:** 10.1155/2011/721608

**Published:** 2011-09-20

**Authors:** Theresa C. Barnes, Marina E. Anderson, Robert J. Moots

**Affiliations:** Department of Rheumatology, Institute of Chronic Disease and Ageing, University of Liverpool and Clinical Sciences Centre, Aintree University Hospital, Longmoor Lane, Liverpool L9 7AL, UK

## Abstract

Interleukin-6 is currently attracting significant interest as a potential therapeutic target in systemic sclerosis (SSc). In this paper, the biology of interleukin-6 is reviewed, and the evidence for interleukin-6 dysregulation in SSc is explored. The role of inteleukin-6 classical and trans signalling pathways in SSc relevant phenomena such as chronic inflammation, autoimmunity, endothelial cell dysfunction, and fibrogenesis is discussed. The existing evidence that interventions designed to block interleukin-6 signalling are of therapeutic relevance in SSc is evaluated.

## 1. Introduction

Systemic sclerosis (SSc) is a connective tissue disease characterised by fibrosis, vasculopathy, and immunological abnormalities. Over recent years, it has become clear that inflammation plays a crucial role in mediating the pathophysiological process underlying SSc, especially early in the disease. Endothelial cell activation and dysfunction are central to the disease pathogenesis, may be driven by a proinflammatory environment, and may result in the generation of a profibrotic phenotype. 

Interleukin-6 (IL-6) is a pleiotropic cytokine. In addition to its role in the acute phase response, IL-6 has diverse roles in driving chronic inflammation, autoimmunity, endothelial cell dysfunction, and fibrogenesis. Therefore, it is currently attracting a great deal of interest in the rheumatology community as a potential therapeutic agent in SSc, a disease which at present lacks treatments directed at the underlying pathogenesis.

Recent evidence has suggested that IL-6 may play important roles in endothelial cell dysfunction and fibrogenesis in this disease, and clinical trials are currently being designed to further explore whether Tocilizumab, a monoclonal antibody directed against the IL-6 receptor, may be of therapeutic benefit to patients with SSc.

## 2. Interleukin-6 Biology

Interleukin-6 biology is complex. Few cells express the interleukin-6 receptor (IL-6R, gp80). This receptor is expressed on hepatocytes, monocytes, B cells, and neutrophils in humans. It is also found on a subset of T cells, but there is evidence that T cells respond to IL-6 predominantly through a process known as trans signalling [1].

Endothelial cells and fibroblasts do not express the IL-6R and are also thought to respond to IL-6 through trans signalling [2]. sIL-6Rs exist in the serum and bind to IL-6 forming an IL-6/sIL-6R complex. Soluble IL-6R (sIL-6R) is produced by two separate mechanisms, firstly by proteolytic cleavage from the surface of neutrophils and secondly by secretion from neutrophils and monocytes of an alternatively spliced version [3–6].

Although the regulation of the proteolytic cleavage of sIL-6R has not been fully elucidated, it is known to be stimulated by C-reactive protein (CRP). Cleavage from the surface of neutrophils, but not monocytes, is also stimulated by chemoattractants (interleukin-8 (IL8), C5a, leukotriene B4 (LTB4), and platelet activating factor (PAF)) [7]. Proteolytic cleavage can occur via a TNF*α*, converting enzyme-like enzyme although this does not account for all of the proteolytic cleavage [7].

We and others have shown that there is an increased concentration of the neutrophil chemoattractant IL-8 in SSc serum [8, 9], which may stimulate the release of sIL-6R from neutrophils. In addition, there are reports in the literature that LTB4 levels are elevated in the bronchoalveolar lavage fluid of patients with SSc lung disease [10], that may also contribute to the generation of sIL-6R.

**Figure 1:**
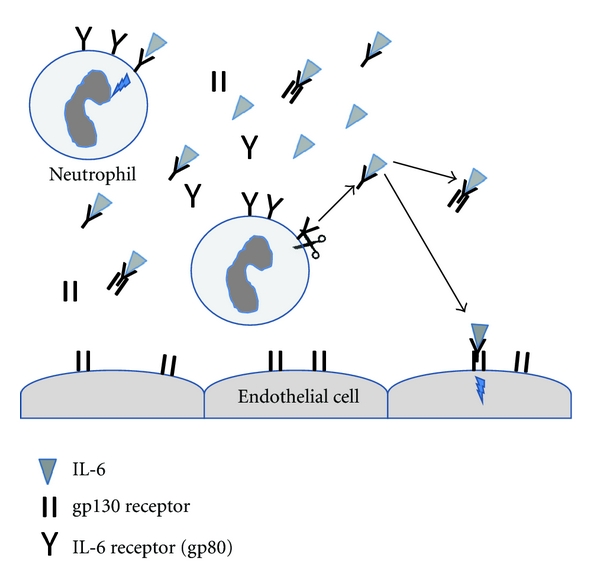
Interleukin-6 trans signalling. IL-6 receptors are expressed on leukocytes including neutrophils, but they are not expressed on tissue-resident cells, for example, endothelial cells. Endothelial cells can respond to IL-6 through the gp130 receptor only when the IL-6 is bound to a soluble IL-6 receptor (sIL-6R). sIL-6Rs are formed by secretion of an alternatively spliced version of the receptor or proteolytic cleavage from the surface of neutrophils. There is also a pool of soluble gp130 (sgp130) which can bind IL-6/sIL6R complexes and prevent them binding to cellular gp130. Therefore, the local concentrations of IL-6, sIL-6R, and sgp130 regulate IL-6 signalling.

The IL6/sIL6R complex can bind to the gp130 receptor, which is expressed ubiquitously on cells including endothelial cells and fibroblasts, to activate the signal transducers and activators of transcription protein 3 (STAT3) signalling pathway [1–11]. Endothelial cell activation via trans signalling results in an increase in the expression of adhesion molecules (intercellular adhesion molecule-1 (ICAM-1), vascular cell adhesion molecule-1 (VCAM-1)), the release of chemokines (IL-8 and monocyte chemotactic protein-1 (MCP-1)), and the release of IL-6 [2–12] (Figure 1).

## 3. Interleukin-6 in Systemic Sclerosis

IL-6 is a cytokine with several potentially important roles in the pathogenesis of SSc. It is elevated in the serum of patients with systemic sclerosis, especially those with diffuse skin involvement and early in the disease course [13, 14]. Immunocytochemistry studies have also demonstrated that IL-6 may be elevated in lesional tissue later in the disease, when other proinflammatory cytokines have dissipated. 

Several other observations further support a role for this interleukin in SSc. Fibroblasts isolated and cultured from the lesional skin of patients with SSc constitutively produce higher levels of IL-6 than nonlesional or healthy donor fibroblasts [15]. This demonstrates the importance of considering local concentrations of cytokines in disease. Serum concentrations may not necessarily reflect local levels of a relevant cytokine at the lesional site. Hence, the use of *in vitro* models to explore local interactions between fibroblasts, endothelial cells, and immune cells, in the presence of locally elevated levels of cytokines, is of particular importance. Stimulated and unstimulated fibroblasts from lesional skin have also been shown to produce increased levels of IL-8 which may be implicated in local release of sIL-6R from neutrophils [16].

Previous research has shown that peripheral blood mononuclear cells from SSc patients, when cultured *in vitro, *produce higher levels of IL-6 and sIL-6R in the culture supernatants than control peripheral blood mononuclear cells, though levels of sgp130 were equivalent [17]. Furthermore, IL-6R levels were increased in the serum of patients with limited cutaneous SSc (lcSSc) compared to controls [18].

IL-6 transcription is under the control of a hypoxic response element via hypoxia-inducible factor-1-*α* (HIF-1-*α*). Measurements taken from the lesional skin of patients have demonstrated a persistent decrease in oxygen tension [19], down the equivalent of 3% O_2_, sufficient to induce HIF-1*α* signalling [19].

In addition, it is important to note that hemodynamic flow may suppress IL-6-induced signalling in endothelial cells [20]. As such flow is dysregulated in SSc, this may play an important role in modulating the effects of IL-6 on endothelial cells in this disease. 

## 4. Interleukin-6 Effects on B Cells

IL-6 also has a profound effect on B cells, promoting plasma cell differentiation and antibody production. This may explain the polyclonal B-cell expansion and hypergammaglobulinaemia which is frequently seen in SSc [11].

B-cell depletion using rituximab (monoclonal antibody directed against CD20) in 9 patients with progressive SSc skin disease, refractory to cyclophosphamide therapy, resulted in a clinical improvement in skin score after 3 months, which persisted up to 36 months. This was paralleled by a decrease in serum IL-6 concentration [21].

## 5. Interleukin-6 and Effects on Inflammation

IL-6 has been implicated in the generation and propagation of chronic inflammation. Initially in acute inflammation, proinflammatory cytokines promote neutrophil accumulation and the release of IL-6. Neutrophils then shed their IL-6Rs in response to chemokines such as IL-8. This promotes differential regulation of chemokine production by endothelial cells, promoting MCP-1 production and decreasing IL-8 production, therefore favouring monocyte accumulation. IL-6 trans signalling also increases the expression of endothelial leukocyte adhesion molecules (VCAM-1, ICAM-1), further promoting leukocyte accumulation [12–22]. In addition, IL-6 may have a role in promoting neutrophil apoptosis and therefore the resolution of acute (nonspecific) inflammation [23, 24]. Others however have reported an antiapoptotic effect of IL-6 on neutrophils [25], while Biffl et al. have shown that the effect depends on the neutrophil concentration [26]. We have been unable to reproduce any IL-6-specific effect on neutrophil apoptosis in our laboratory at concentrations of IL-6 ranging from 0.1 to 100 ng/mL (personal communication Helen Wright). 

Conversely, IL-6 reportedly rescues T cells from apoptosis, which promotes a chronic inflammatory cell infiltrate [27–30]. IL-6 trans signalling also promotes the release of IL-6 from fibroblasts and endothelial cells in a positive autocrine feedback system. Therefore, it can be envisaged that IL-6 may have a role in propagating chronic inflammation, such as that seen in SSc. This is in keeping with immunocytochemical experiments which demonstrate that IL-8 and IL-6 are overexpressed in the lesional skin of patients with SSc, though in different patterns: the overexpression of IL-8 is associated with early disease (<1 yr), whereas IL-6 overexpression is associated with later disease [31]. 

IL-6 has also been implicated in autoimmunity. Evidence from patients with Crohn's disease indicates that autoreactive T cells are resistant to apoptosis due to protection by IL-6 trans signalling via the STAT3 signalling pathway [32]. IL-6 inhibits a Na^2^+/K+ ATPase which regulates antigen internalisation and antigen presentation by dendritic cells to T cells, which may promote presentation of autoantigens [33, 34]. Finally, according to Matzinger's “danger theory,” naïve T cells die if they receive a signal from proper antigen presentation that is not followed up by ligation of CD40 [35]. There is evidence that IL-6/sIL-6R complex can inappropriately substitute for this second signal and therefore lead to the persistence of autoreactive T cells [36]. Furthermore, autoimmune phenomena increase with age, in concert with an age-related increase in sIL-6R shedding [37]. Lissilaa et al. explored the role of IL-6 in the collagen-induced arthritis (CIA) and antigen-induced arthritis (AIA) models of autoimmune inflammatory arthritis. Using antibodies which specifically blocked classical IL-6 signalling and trans signalling pathways, they discovered that the classical IL-6 pathway was both necessary and sufficient for the development of pathogenic Th17 T cells which are implicated in autoimmunity and for the generation of antitype II collagen IgG responses which are associated with disease manifestations in the CIA model. They also demonstrated in the AIA model that IL-6 trans signalling was responsible for driving local inflammatory responses [38]. SSc is a disease associated with autoimmune phenomena. Many different autoantibodies are found in SSc (see Table 1), and the autoantibody profile in many cases correlates with clinical manifestations. There is, however, no convincing evidence for a direct role for autoantibodies in pathogenesis though some investigators have reported that antiendothelial cell antibodies, found in a proportion of patients, are associated with endothelial cell activation [39, 40].

## 6. Interleukin-6 and Effects on Fibrogenesis

Fibroblasts from patients with SSc are phenotypically unique. When isolated and cultured *in vitro *they continue to produce an excess of collagen [42, 43]. IL-6 is a profibrogenic cytokine. It has been shown to either increase or decrease fibroblast proliferation, increase fibroblast collagen, glycosaminoglycan, and tissue inhibitor of metalloproteinases-1 (TIMP-1) synthesis, and increase MCP-1 and IL-6 production [43–48]. IL-6 regulates the expression of vascular endothelial growth factor (VEGF), an important mediator of angiogenesis and fibrosis which is elevated in patients with SSc [49].

One case series has indicated that the use of tocilizumab, which blocks IL-6 trans signalling, in 2 patients with diffuse cutaneous SSc (dcSSc), one with renal involvement and the other with lung fibrosis, resulted in a decrease in skin thickening as measured by Rodnan skin score and Vesmeter (which measures viscoelasticity or hardness of the skin). In addition, skin biopsies taken before and after tocilizumab treatment indicated a reduction in collagen [50].

## 7. Interleukin-6 and Effects on Endothelial Cell Activation

Endothelial activation is thought to be central to the pathogenesis of SSc. There is also evidence for increased endothelial cell apoptosis though corroborative *in vivo* evidence for this is lacking [51]. The University of California at Davis line 200 chicken, an animal model for SSc, shows evidence of early endothelial cell apoptosis, preceding the inflammatory cell infiltrate and the development of fibrosis [39–52]. 

Serum markers of endothelial cell activation, for example, von Willebrand factor (vWF), sICAM-1, and sE-selectin are elevated in the serum of patients with SSc and appear to correlate with disease activity [53–55].

Previous studies have shown a role for IL-6 in endothelial cell activation. Endothelial cell activation via trans signalling results in an increase in the expression of adhesion molecules (ICAM-1, VCAM-1), the release of chemokines (IL-8 and MCP), and the release of IL-6 [2–12].

We have recently shown that SSc serum, in the presence of neutrophils, is capable of increasing endothelial cell activation and apoptosis in an IL-6-dependent manner [56]. It is postulated that in this circumstance the neutrophils are acting as donors of IL-6R. In our studies, spiking pooled control serum with IL-6 resulted in increased endothelial cell apoptosis and E-selectin expression in the presence of neutrophils, mimicking the effects of SSc serum. Complement inactivation did not abrogate the effects of SSc serum, neither did the addition of catalase to mop up reactive oxygen species. The serine protease inhibitor AEBSF partially blocked the effects of SSc serum on endothelial cell apoptosis but did not significantly affect the activation of endothelial cells by SSc serum [56]. Strategies to remove or block the effects of IL6 in SSc serum including immunodepletion of IL6 and the addition of an anti-IL6 blocking antibody reversed the effects of SSc serum on endothelial cell activation and apoptosis [56]. Most significantly, however, sgp130 which specifically blocks IL6 trans signalling abrogated the effects of SSc serum [56].

## 8. Conclusion

IL-6 blockade and specifically the blockade of IL-6 trans-signalling may have merit in the treatment of SSc, a disease that so far lacks treatment options directly targeting the pathogenic mechanism. IL-6 trans signalling is specifically implicated in driving local inflammation and inducing endothelial and fibroblast responses, and therefore targeting this IL-6 signalling pathway may be most profitable in SSc. However, SSc also has important and possibly pathogenic autoimmune phenomena, and targeting the classical IL-6 signalling pathway may be necessary in order to influence this important aspect of the disease. The currently available drug Tocilizumab targets both the classical and the trans signalling pathways. Other agents are in development which specifically block trans signalling, and they may be useful in mouse models of SSc to delineate which signalling pathway is most important for this disease. 

IL-6 is increased in the serum of patients with SSc, especially in early dcSSc. In addition, it is also found in immunohistochemistry samples in both early and late disease and in both dcSSc and lcSSc. Fibroblasts and monocytes isolated from SSc patients autonomously produce IL-6 *in vitro*. 

Early, small-scale nonrandomised controlled trials point to an important role for IL6 in SSc. B-cell depletion results in a decrease in serum IL-6 levels, reflected in a simultaneous reduction in skin score. More importantly, blocking IL-6 trans signalling with Tocilizumab has resulted in an improvement in skin score in 2 patients with diffuse disease. These data firmly establish IL-6 as an attractive candidate therapeutic target, especially in terms of preventing fibrosis. 

However, in addition, new and exciting data imply that IL-6 has a role in the endothelial and inflammatory manifestations of this disease, which may make it a potential target in a much broader range of SSc patients with active vascular or inflammatory (e.g., joint) disease but relatively little fibrosis. Studies are being designed to address these important questions; the results are eagerly awaited.

## Figures and Tables

**Table 1:** Systemic sclerosis-associated autoantibodies, potentially pathogenic antibodies which have been described in a proportion of patients with systemic sclerosis. Reviewed in [41]. ECM: extracellular matrix.

Autoantibody	*In vitro* activity
Antiendothelial cell	Endothelial cell apoptosis
Antifibrillin 1	Fibroblast activation, increased ECM production
Antimatrix metalloproteinase	Prevent degradation of the ECM
Anti-PDGFR	Induce collagen 1 production Convert fibroblasts to myofibroblasts
Antifibroblast	Increased expression of ICAM and IL-6
Anti-HSP47	Not known
